# Data Discovery and Anomaly Detection Using Atypicality for Real-Valued Data

**DOI:** 10.3390/e21030219

**Published:** 2019-02-26

**Authors:** Elyas Sabeti, Anders Høst-Madsen

**Affiliations:** 1 Department of Computational Medicine and Bioinformatics, University of Michigan, NCRC 10-A108, 2800 Plymouth Rd, Ann Arbor, MI 48109-2800, USA; 2 Department of Electrical Engineering, University of Hawaii at Manoa, Honolulu, HI 96822, USA; 3 Shenzhen Research Institute of Big Data, Shenzhen 518172, China

**Keywords:** atypicality, minimum description length, big data, codelength

## Abstract

The aim of using atypicality is to extract small, rare, unusual and interesting pieces out of big data. This complements statistics about typical data to give insight into data. In order to find such “interesting” parts of data, universal approaches are required, since it is not known in advance what we are looking for. We therefore base the atypicality criterion on codelength. In a prior paper we developed the methodology for discrete-valued data, and the current paper extends this to real-valued data. This is done by using minimum description length (MDL). We develop the information-theoretic methodology for a number of “universal” signal processing models, and finally apply them to recorded hydrophone data and heart rate variability (HRV) signal.

## 1. Introduction

A central question in the era of “big data” is what to do with the enormous amount of information. One possibility is to characterize it through statistics, e.g., averages, or classify it using machine learning, in order to understand the general structure of the overall data. The perspective in this paper is the opposite, namely that most of the value in the information—in some applications—is in the parts that deviate from the average, that are unusual, atypical. Think of art: The valuable paintings or writings are those that deviate from the norms and brake the rules, that are atypical. Or groundbreaking scientific discoveries, which find new structure in data. Finding such unusual data is often done by painstaking human evaluation of data. The goal of our work is to find practical, automatic methods for localizing atypical parts of data.

When searching for atypical data, a key characteristic is that we do not know what we are looking for, we are looking for the “unknown unknowns”. We therefore need universal methods. In the paper [1] we developed a methodology, atypicality, that can be used to discover such data. The basic idea is that if some data can be encoded with a shorter codelength in itself, i.e., with a universal source coder, rather than using the optimum coder for typical data, then it is atypical. The purpose of the current paper is to generalize this to real-valued data. Lossless source coding does not generalize directly to real-valued data. Instead we can use minimum description length (MDL). In the current paper we develop an approach to atypicality based on MDL, and show its usefulness on a real dataset.

In this section before an extensive literature review of detection problems, we first describe the concepts of atypicality and how this framework can be used for data discovery. This arrangement is essential in order to compare the atypicality with the state of the art methods.

### 1.1. Anomaly Detection and Data Discovery Based on Description Length

A common way to define an outlier or anomaly in data is a sample that does not fit the statistics of typical data [2], e.g., if typical data is described by a pdf $f_{T}\left(x\right)$, and if $f_{T}\left(x\right)<\tau$ for some threshold τ then *x* is an outlier. In this paper we approach the problem of anomaly detection, and in particular data discovery, from a different point of view. We consider sequences of data $x^{l}$, and say that a sequence of data $x^{l}$ is atypical if there is some alternative model that ’fits’ the data better than the typical model. This point of view has been considered before in anomaly detection, e.g., [3]. Given a typical probability distribution, data that is unlikely could simply be, well, outliers, e.g., faulty measurements, and not of much interest in itself. Requiring data to fit an alternative model gives an indication that there is some interesting, new relationship in the data. We therefore think of this approach going beyond simply finding anomalous data, to finding interesting data, i.e., data discovery.

In our paper [1] we used universal source coding for anomaly detection; in [3,4,5] the authors used a type of universal empirical histogram. This kind of methodology is feasible when data is discrete. However, real-valued data is too rich for such universal descriptions. Models for real-valued data is almost always given as parametric models, either directly or indirectly. Our approach to atypicality for real-valued data, in the absence of universal coders, is to consider multiple ‘universal’ real-valued models given by parametric models. For example, it is well-known [6] that by the Wold decomposition (almost) all Gaussian stationary processes can be described in terms of a linear prediction model. Wavelets are also good for compressing (lossily) many signals and images. One can therefore expect these will also work well as alternative models. Most modeling and compression are based on a second order analysis, and therefore fit with Gaussian models. One could be interested in also finding atypical data that does not fit a Gaussian model; however, apart from iid (independently, identically distributed) models (similar to [3]), this is difficult to do, so the richness of non-Gaussian models is limited. We will therefore focus on Gaussian models in this paper; notice, however, this is not a limitation of atypicality, we have considered non-Gaussian models in [7].

Consider an atypicality setup where the typical model is given by a probability density function (pdf) $f_{T}\left(x^{l}\right)$ and the atypical model is given by f(x|θ) with θ unknown. Asking if the atypical model is better can be thought of simply as a generalized likelihood ratio test (GLRT) hypothesis test [8].

$$\frac{\min_{\theta }f\left(x^{l}|\theta \right)}{f_{T}\left(x^{l}\right)}\geq \tau .$$

However, in atypicality we would like to test the sequence with respect to a large class of alternative hypotheses—even the class of linear prediction models is infinite. So, assume we have a finite or countable infinite set of model classes $M_{i}$ with corresponding pdfs $f_{i}\left(x|\theta_{i}\right)$. A test could then be

(1) $$\frac{\min_{i}\min_{\theta_{i}}f_{i}\left(x^{l}|\theta_{i}\right)}{f_{T}\left(x^{l}\right)}\geq \tau .$$

However, this is clearly not very useful. More and more complex model will fit data better and better [9], so that the false alarm probability will be very large—model complexity has to be taken into account. One way to do this through Bayesian statistics assigning prior probabilities to both models and parameters, ending up in the test

(2) $$\frac{P_{A}\sum_{i}P\left(M_{i}\right)\int f_{i}\left(x^{l}|\theta_{i}\right)f_{i}\left(\theta_{i}\right)d\theta_{i}}{\left(1-P_{A}\right)f_{T}\left(x^{l}\right)}\geq 1$$

where $P_{A}$ is the probability of a sequence being atypical and $P(M_{i})$ the probability of an alternative model $M_{i}$. The issue is that using (2) requires choosing a lot of prior distributions and being able to calculate marginal distributions $\int f_{i}\left(x^{l}|\theta_{i}\right)f_{i}\left(\theta_{i}\right)d\theta_{i}$. As explained in for example (3.4–3.5 [9]), these are not easy problems to tackle. Priors are often dictated by the need for the integral to be calculable, rather than actual prior information, and it still leaves parameters unknown (‘hyperparameters’). In addition, choosing prior distributions is anathema to the central idea of looking for unknown data in big data. The whole point is that we know very little about the data we are looking for.

This is where we can use description length. Suppose at first that data is discrete-valued. To each sequence $x^{l}$ we assign a codeword $c(x^{l})$ with length $L(x^{l})$. The codewords have to be prefix free and the lengths therefore have to satisfy the Kraft inequality [10]: $\sum_{x^{l}}2^{-L(x^{l})}\leq 1$. If we let $p\left(x^{l}\right)=2^{-L(x^{l})}$ this defines a (sub)probability on the data, which can be used in a hypothesis test. One can think of description length and coding as a method to find probabilities. There is a key advantage in using description length, as explained in the following. In decoding, a decoder reads a sequence of bits sequentially and turns this into a copy of the source sequence; the codes must be prefix-free. Key here is that in the current step the decoder can only use what is decoded in prior steps. Therefore, when the source sequence is encoded, the encoder cannot use future samples to encode the current sample. We call this ’the principle of sequentiality’. It is the Kraft inequality in reverse: In one direction, as above, we can use the Kraft inequality to verify that a set of codelengths gives valid codes. In the other direction, when codes are decodable (in the pre-fix free sense), they must satisfy the Kraft inequality, and the corresponding probabilities must therefore be valid. An example is Lempel-Ziv coding [10,11,12], which does not explicitly rely on probabilities. It gives valid codewords because the coding is decodable with a sequential decoder.

To generalize the coding approach to real-valued data, lossless coding is needed. One can notice that lossless coding of real-valued data is used in many applications, for example lossless audio coding [13]. However, direct encoding of the reals represented as binary numbers, such as done in lossless audio coding, makes the methods too dependent on data representation rather than the underlying data. Instead we will use a more abstract model of (finite-precision) reals. We will assume a fixed point representation with a (large) finite number, *r*, bits after the period, and an unlimited number of bits prior to the period as in [14]. Assume that the actual data is distributed according to a pdf f(x). Then the number of bits required to represent *x* is given by

(3) $$\begin{array}{cc} L(x) & =-\log\int_{x}^{x+2^{-r}}f\left(t\right)dt\approx -\log\left(f\left(x\right)2^{-r}\right)\\ & =-\log(f(x))+r. \end{array}$$

As we are only interested in comparing codelengths the dependency on *r* cancels out. Suppose we want to decide between two models $f_{1}\left(x\right)$ and $f_{2}\left(x\right)$ for data. Then we decide $f_{1}\left(x\right)$ if $\lim_{r\to \infty }-\log\int_{x}^{x+2^{-r}}f_{1}\left(t\right)dt+\log\int_{x}^{x+2^{-r}}f_{2}\left(t\right)dt>0$, which is $-\log f_{1}\left(x\right)>-\log f_{2}\left(x\right)$. Thus, for the typical codelength we can simply use $L_{T}\left(x\right)=-\log f_{T}\left(x\right)$. One can also argue for this codelength more fundamentally from finite blocklength rate-distortion in the limit of low distortion [15], which makes it more theoretically well-founded. Notice that this codelength is not scaling invariant:

(4) $$\begin{array}{cc} y & =ax+b\\ L_{t}\left(y\right) & =-\log f(x)+\log|a| \end{array}$$

which means care has to be taken when transforms of data are considered. To code the atypical distributions, as the decoder does not know the values of the parameters, both data and parameters in parametric models have to be encoded for a decoder to be able to decode; this was the starting point in the original paper on MDL [14]. One could also use a Bayesian distribution $\int f_{i}\left(x^{l}|\theta_{i}\right)f_{i}\left(\theta_{i}\right)d\theta_{i}$ from (2), which does not solve the issues with using Bayes. Instead we can use the principle of sequentiality of coding as follows. We replace $\int f_{i}\left(x^{l}|\theta_{i}\right)f_{i}\left(\theta_{i}\right)d\theta_{i}$ in (2) with a codelength based on Rissanen’s predictive MDL [16].

(5) $$L_{i}\left(x^{l}\right)=-\sum_{n=0}^{l-1}\log f_{i}\left(x_{n+1}|{\hat{\theta }}_{i}\left(x^{n}\right)\right)$$

where $\hat{\theta }\left(x^{i}\right)$ is the maximum likelihood estimate of the parameter. Since this is sequentially decodable, it gives a valid codelength, and hence probability, without any prior distribution on θ. It does not work for the first sample, as there is no estimate. Instead we encode $x_{1}$ with a default distribution. In general application of MDL choice of the default distribution can be tricky, but for atypicality we have a good default distribution: The typical distribution, giving the codelength

(6) $$L_{i}\left(x^{l}\right)=-\sum_{n=1}^{l-1}\log f_{i}\left(x_{n+1}|{\hat{\theta }}_{i}\left(x^{n}\right)\right)-\log f_{T}\left(x_{1}\right).$$

Notice that default distribution is the same for all models $M_{i}$: we do not have to choose a prior for each model. There are no prior assumptions involved, since we use the typical distribution. We still need the probabilities $P(M_{i})$; here we can use Rissanen’s universal coder of the integers [14]. The codelength for an integer *i* is $c+\log^{*}i$ where *c* is a normalization constant in the Kraft inequality [14] and $\log^{*}l=\log l+\log\log l+\cdots$ with the sum continuing as long as the log is defined. We order the models according to complexity and encode the ordinal of a model. The description length test for the sequence $x^{l}$ to be atypical then becomes

(7) $$\begin{array}{c} -\log\left(\sum 2^{-L_{i}\left(x^{l}\right)-\log^{*}i-c}\right)-\log P_{A}\\ \leq -\log f_{T}\left(x^{l}\right)-\log\left(1-P_{A}\right). \end{array}$$

The appeal of coding becomes even more clear when we search for atypical subsequences of long sequences. Using coding this can be done as follows. The coder uses a special header, a codeword not a prefix of any codeword used for the actual data, to denote the start of a subsequence—the decoder will now know it needs to use the atypical decoder. It also encodes the length of the atypical subsequence using Rissanen’s universal coder for the integers [14], adding $c+\log^{*}l$ to the code length, so that the decoder knows when to switch back to the typical coder. The whole sequence is sequentially decodable, thus has a valid probability, and we know from [1] that this gives a valid criterion, at least for iid sequences, in the sense that not the whole sequence will be classified as atypical; the key is the insistence on decodability. It would be difficult to do this directly using Bayesian analysis, as we would have to develop probability distributions for the total sequence for every combination of atypical subsequences in the long sequence. To be precise, for every set of potential subsequences $S=\{x_{s_{1}}^{e_{1}},x_{s_{2}}^{e_{2}},\ldots \}$ we would have to calculate $p\left(x^{l}|S\right)p\left(S\right)$, and then choose the S giving the largest probability, i.e., MAP.

To understand how (7) avoids the problem overfitting of (1), we notice that asymptotically for large *l* by [16]

(8) $$L_{i}\left(x^{l}\right)\approx f\left(x^{l}|\hat{\theta }\left(x^{l}\right)\right)+\frac{k}{2}\log l$$

where *k* is the number of parameters in θ; this is true for many MDL and Bayesian methods, including Rissanen’s original approach [14]. Because (8) penalizes models with many parameters, overfitting is avoided even if we consider an infinite collection of models. While (8) is often used for model selection, it is not accurate enough for our purposes, and we use (5) directly. However, (8) is useful for discussion and analysis.

The above approach can be seen as a generalization to real-valued data of the approach in [1]

**Definition** **1.** *A sequence is atypical if it can be described (coded) with fewer bits in itself rather than using the (optimum) code for typical sequences*.

There is a further difference from Bayes (2), which is more philosophical than computational and practical. When we describe the problem as a hypothesis test problem as in (2), we are asking which hypothesis is correct (which is also the basis of Bayesian model selection [9]). However, in stating the problem as a description length problem, we are just asking if we can find a shorter description length, not if a model is correct. By considering a very large class of alternative models (most pronounced when we use universal source coding), none might fit very well, none might be even close to the actual model, but we might find one that fits better than the typical model, and that is sufficient for a sequence to be atypical. We have no idea how atypical data might look like, so we cast a very wide net.

### 1.2. Alternative Approaches

Atypicality has many applications: Anomaly detection, outlier detection, data discovery, novelty detection, transient detection, search for ‘interesting’ data etc. What all of these applications have in common is that we seek data that is unusual in some way, and atypicality is a general method for finding such data. Each of these applications have specific alternative methods, and we will discuss atypicality compared to other approaches in some of these applications.

There is a very large existing literature on anomaly detection [17,18,19,20,21,22,23,24,25,26]; The paper [17] gives an overview until 2009. What is characteristic of all methods, as far as we know, is that they look for data that do not fit the characteristics of normal data, either statistically or according to some other measure. From [17]: “At an abstract level, an anomaly is defined as a pattern that does not conform to expected normal behavior.” Atypicality takes a different approach. Atypicality looks for data where an alternative model fits the the data better. Atypicality will still find the first type of anomalies according to [17], but it will also be able to find a wider, more subtle class of anomalies. As a simple example, suppose the normal data is iid Gaussian with zero mean and variance $\sigma^{2}$. The anomalous data is also Gaussian with zero mean and variance $\sigma^{2}$, but the noise is colored. This is in no way anomalous according to the definition in [17]. However, by coding data with a linear predictive coder (see Section 3.2 later) atypicality will detect the anomalous sequence. In [27] we in fact prove that atypicality is exactly optimum for discrete data in the class of finite state machines. While we do not have a similar theorem for real-valued data, this indicates the advantages of atypicality for anomaly detection.

Another advantage of atypicality is that it can straightforwardly be applied to data of unknown/variable length, as discussed in Section 1.1. All existing anomaly detection algorithms we know of use fixed windows, so they cannot make decisions between long, slightly unusual sequences, and short, very unusual sequences; atypicality can. On the other hand, atypicality cannot find single, anomalous samples—outliers: To be able to find a new model for anomalous data, it needs a collection of samples. For this kind of application, more traditional methods must be used.

A type of detection problem closely related to anomaly detection is transient detection [28,29,30,31,32,33,34]. In many signal processing applications, it is of interest to detect short-duration statistical changes in observed data. For a parametric class of probability distribution $\left\{f\left(x|\theta \right):\theta \in \Theta \right\}$ and for an unknown $n_{s}$ and $n_{d}$ the following two hypotheses are considered:

$$\begin{array}{cc} H_{0}: & x_{1}^{l}∼f\left(x|\theta_{0}\right)\\ H_{1}: & x_{1}^{n_{s}-1}∼f\left(x|\theta_{0}\right),\;x_{n_{s}}^{n_{d-1}}∼f\left(x|\theta_{1}\right),\;x_{n_{d}}^{l}∼f\left(x|\theta_{0}\right). \end{array}$$

If $\theta_{0}$ and $\theta_{1}$ are known, the Page test is optimal for this in the sense that by using a GLRT; given an average wait between false alarms, it minimizes the worst-case average delay to detection [31]. However in many applications, there is either no information about $\theta_{1}$ or it varies from one transient signal to another. In this case, it is shown that Variable Threshold Page (VTP) gives a reliable result [29,31]. There are also other approaches of transient detection based on Nuttall’s power-law detector that are often used in the literature [29,30]. Other methods are [32,33,34]. In general atypicality will outperform this methods since it not only allows a more comprehensive class of models, but also it can take advantage of various powerful signal processing methods such as filterbanks and linear prediction to find transient signals with various statistics.

Finally, we will mention change point detection and quickest change detection [35,36,37,38,39,40,41,42]. The goal here is to find a point in time where the distribution of data changes from one to another. The difference from atypicality is that in atypicality, subsequences have both a start and end point. In principle one could use atypicality for change point detection, but since it is not optimized for this application, the comparison is not that relevant, and atypicality might not perform well. We refer to [35,36] for how to use MDL for change point detection.

## 2. Minimum Description Length Methods

**Figure 1:**
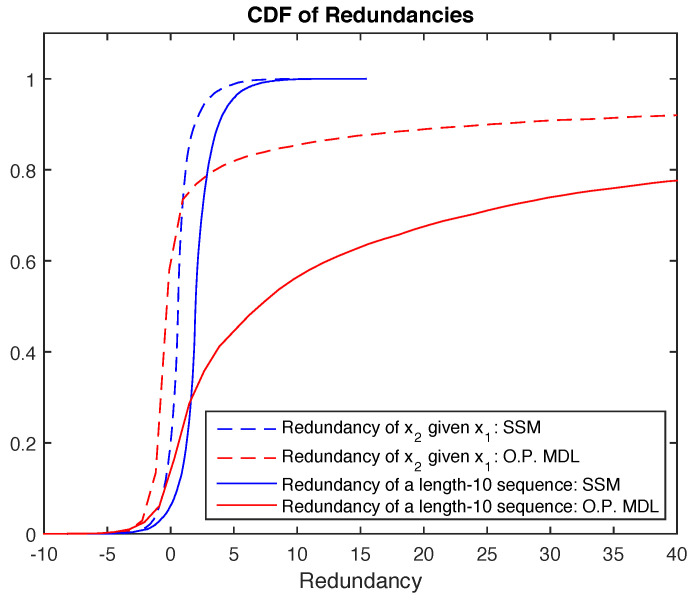
Redundancy comparison between ordinary predictive minimum description length (O.P. MDL) and our proposed sufficient statistic method for μ=0 and $\sigma^{2}=4$.

Above we have argued for using (5) as a codelength. The issue with this method is how to initialize the recursion. In (6) this is solved by using the typical distribution for the first sample, but in general, with more than one parameter, ${\hat{\theta }}_{i}\left(x^{i}\right)$ may not be defined until *i* becomes larger than 1. The further issue is that even when $\hat{\theta }\left(x^{i}\right)$ *is* defined, the estimate might be poor for small *i*, and using this in (5) can give very long codelengths, see Figure 1 below.

Our solution to the first issue is to encode with increasingly complex models as *i* increases; we therefore only have to use the default distribution for the very first sample. Since we are not interested in finding a specific model, this is not problematic in atypicality. Our solution to the second issue is rather than using the ML estimate for encoding as though it is the actual parameter value, we use it as an uncertain estimate of θ. We then take this uncertainty into account in the codelength. This is similar to the idea of using confidence intervals in statistical estimates [43]. Below we introduce two methods using this general principle. This is different to the sequentially normalized maximum likelihood method [44], which modifies the encoder itself.

### 2.1. Sufficient Statistic Method (SSM)

As explained above, our approach to predictive MDL is to introduce uncertainty in the estimate of θ. Our first methodology is best explained through a simple example. Suppose our model is $N(\mu ,\sigma^{2})$, with σ known. The average ${\bar{x}}_{n}$ is the ML estimate of μ at time *n*. We know that

$${\bar{x}}_{n}=\mu +z,\;z∼N\left(0,\frac{\sigma^{2}}{n}\right).$$

We can re-arrange this as

$$\mu ={\bar{x}}_{n}-z.$$

Thus, *given* ${\bar{x}}_{n}$, we can think of μ as random $N\left({\bar{x}}_{n},\frac{\sigma^{2}}{n}\right)$. Now

$$x_{n+1}=\mu +z_{n+1}∼N\left({\bar{x}}_{n},\sigma^{2}+\frac{\sigma^{2}}{n}\right)$$

which we can use as a coding distribution for $x_{n+1}$. This compares to $N\left({\bar{x}}_{n},\sigma^{2}\right)$ that we would use in traditional predictive MDL. Thus, we have taken into account that the estimate of μ is uncertain for *n* small. The idea of thinking of the non-random parameter μ as random is very similar to the philosophical argument for confidence intervals [43].

In order to generalize this example to more complex models, we take the following approach. Suppose $t(x^{n})$ is a *k*-dimensional sufficient statistic for the *k*-dimensional θ∈Θ. Also suppose there exists some function s and a *k*-dimensional (vector) random variable Y independent of θ so that

(9) $$t\left(x^{n}\right)=s\left(Y,\theta \right).$$

We now assume that for every (t,Y) in their respective support, (9) has a solution for θ∈Θ so that we can write

(10) $$\theta =r(Y,t\left(x^{n}\right)).$$

The parameter θ is now a random variable (assuming r is measurable, clearly) with a pdf $f_{x^{n}}\left(\theta \right)$ This then gives a distribution on $x_{n+1}$, i.e.,

(11) $$\begin{array}{cc} f(x_{n+1}|x^{n}) & =\int f\left(x_{n+1}|\theta \right)f_{x^{n}}\left(\theta \right)d\theta . \end{array}$$

The method has the following property:

**Theorem** **1.** *The distribution of $x_{n+1}$ is invariant to arbitrary parameter transformations.*

This is a simple observation from the fact that (11) is an expectation, and that when θ is transformed, the distribution according to (10) is also transformed with the same function.

One concern is the way the method is described. Perhaps we could use different functions s and r and get a different result? In the following we will prove that the distribution of θ is independent of which s and r are used.

It is well-known [6,10] that if the random variable *X* has CDF *F*, then U=F(X) has a uniform distribution (on [0,1]). Equivalently, $X=F^{-1}\left(U\right)$ for some uniform random variable *U*. We need to generalize this to *n* dimensions. Recall that for a continuous random variable [6]

$$\begin{array}{c} F_{i|i-1,\ldots ,1}\left(x_{i}|x_{i-1},\ldots x_{1}\right)=\int_{-\infty }^{x_{i}}f\left(t|x_{i-1},\ldots ,x_{1}\right)dt\\ =\frac{1}{f(x_{i-1},\ldots ,x_{1})}\int_{-\infty }^{x_{i}}f\left(t,x_{i-1},\ldots ,x_{1}\right)dt \end{array}$$

whenever $f(x_{i-1},\ldots ,x_{1})\neq 0$. As an example, let n=2. Then the map $\left(X_{1},X_{2}\right)\mapsto \left(F_{1}\left(X_{1}\right),F_{2|1}\left(X_{2},X_{1}\right)\right)$ is a map from $R^{2}$ onto ${\left[0,1\right]}^{2}$, and $\left(F_{1}\left(X_{1}\right),F_{2|1}\left(X_{2},X_{1}\right)\right)$ has uniform distribution on ${\left[0,1\right]}^{2}$. Here $F_{1}\left(X_{1}\right)$ is continuous in $X_{1}$ and $F_{2|1}\left(X_{2},X_{1}\right)$ is continuous in $X_{2}$

We can write $X_{1}=F_{1}^{-1}\left(U_{1}\right)$. For fixed $x_{1}$ we can also write $X_{2}=F_{2|1}^{-1}\left(U_{2}|x_{1}\right)$ for those $x_{1}$ where $F_{2|1}$ is defined, and where the inverse function is only with respect to the parameter before |. Then

$$\left[\begin{array}{c} X_{1}\\ X_{2} \end{array}\right]=\left[\begin{array}{c} F_{1}^{-1}\left(U_{1}\right)\\ F_{2|1}^{-1}\left(U_{2}|F_{1}^{-1}\left(U_{1}\right)\right) \end{array}\right]≜{\overset{ˇ}{F}}^{-1}\left(U_{1},U_{2}\right).$$

This gives the correct joint distribution on $\left(X_{1},X_{2}\right)$: The marginal distribution on $X_{1}$ is correct, and the conditional distribution of $X_{2}$ given $X_{1}$ is also correct, and this is sufficient. Clearly ${\overset{ˇ}{F}}^{-1}$ is not defined for all $U_{1},U_{2}$; the relationship should be understood as being valid for almost all $\left(X_{1},X_{2}\right)$ and $\left(U_{1},U_{2}\right)$. We can now continue like this for $X_{3},X_{4},\ldots ,X_{n}$. We will state this result as a lemma

**Lemma** **2.** *For any continuous random variable X there exists an n-dimensional uniform random variable U, so that $X={\overset{ˇ}{F}}^{-1}\left(U\right)$.*

**Theorem** **2.** *Consider a model $t=s_{1}\left(Y_{1};\theta \right),$ with $\theta =r_{1}\left(Y_{1};t\right)$ and an alternative model $t=s_{2}\left(Y_{2};\theta \right),$ with $\theta =r_{2}\left(Y_{2};t\right).$ We make the following assumptions:*

*1.*  *The support of t is independent of **θ** and its interior is connected.*
*2.*  *The extended CDF ${\overset{ˇ}{F}}_{i}$ of $Y_{i}$ is continuous and differentiable.*
*3.*  *The function $Y_{i}\mapsto s_{i}\left(Y_{i};\theta \right)$ is one-to-one, continuous, and differentiable for fixed **θ**.*

*Then the distributions of **θ** given by $r_{1}$ and $r_{2}$ are identical.*

**Proof.**  By Lemma 2 write $Y_{1}=F_{1}^{-1}\left(U_{1}\right)$, $Y_{2}=F_{2}^{-1}\left(U_{2}\right)$. Let *u* be the *k*-dimensional uniform pdf, i.e., u(x)=1 for $x\in {\left[0,1\right]}^{k}$ and 0 otherwise, and let $Y_{i}=s_{i}^{-1}\left(t;\theta \right)$ denote the solution of $t=s_{i}\left(Y_{i};\theta \right)$ with respect to $Y_{i}$, which is a well-defined due to Assumption 3. We can then write the distribution of t in two ways as follows ([6]), due to the differentiability assumptions

$$\begin{array}{cc} f(t;\theta ) & =u(F_{1}\left(s_{1}^{-1}\left(t;\theta \right)\right)\left|\frac{\partial F_{1}(s_{1}^{-1}\left(t;\theta \right)}{\partial t}\right|\\ & =u(F_{2}\left(s_{2}^{-1}\left(t;\theta \right)\right)\left|\frac{\partial F_{2}(s_{2}^{-1}\left(t;\theta \right)}{\partial t}\right|. \end{array}$$

Due to Assumption 1 we can then that conclude $\frac{\partial F_{1}(s_{1}^{-1}\left(t;\theta \right)}{\partial t}=\frac{\partial F_{2}(s_{2}^{-1}\left(t;\theta \right)}{\partial t}$, or

$$F_{1}(s_{1}^{-1}\left(t;\theta \right)=F_{2}\left(s_{2}^{-1}\left(t;\theta \right)\right)+k\left(\theta \right).$$

But both $F_{1}$ and $F_{2}$ have range ${\left[0,1\right]}^{k}$, and it follows that k(θ)=0. Therefore

$$t=s_{1}\left(F_{1}^{-1}\left(U\right);\theta \right)=s_{2}\left(F_{2}^{-1}\left(U\right);\theta \right).$$

If we then solve either for θ as a function of U (for fixed t), we therefore get exactly the same result, and therefore the same distribution. □

The assumptions of Theorem 2 are very restrictive, but we believe they are far from necessary. In [45] we proved uniqueness in the one-dimensional case under much weaker assumptions (e.g., no differentiability assumptions), but that proof is not easy to generalize to higher dimensions.

**Corollary** **3.** *Let $t_{1}\left(x^{n}\right)$ and $t_{2}\left(x^{n}\right)$ be equivalent sufficient statistic for **θ**, and assume the equivalence map is a diffeomorphism. Then the distribution on **θ** given by the sufficient statistic approach is the same for $t_{1}$ and $t_{2}$.*

**Proof.**  We have $t_{1}=s_{1}\left(Y_{1},\theta \right)$ and $t_{2}=s_{2}\left(Y_{2},\theta \right)$. By assumption, there exists a one-to-one map *a* so that $t_{1}=a\left(t_{2}\right)$, thus $t_{1}=a\left(s_{2}\left(Y_{2},\theta \right)\right)$. Since the distribution of θ is independent of how the problem is stated, $t_{1}$ and $t_{2}$ gives the same distribution on θ. □

### 2.2. Normalized Likelihood Method (NLM)

The issue with the sufficient statistic method is that a sufficient statistic of the same dimension of the parameter vector can be impossible to find. We will therefore introduce a simpler method. Let the likelihood function of the model be $f(x^{l}|\theta )$. For a fixed $x^{l}$ we can consider this as a ‘distribution’ on θ; the ML estimate is of course the most likely value of this distribution. To account for uncertainty in the estimate, we can instead try use the total $f(x^{l}|\theta )$ to give a distribution on θ, and then use this for prediction. In general $f(x^{l}|\theta )$ is not a probability distribution as it does not integrate to 1 in θ. We can therefore normalize it to get a probability distribution

(12) $$f_{x^{l}}\left(\theta \right)=\frac{f(x^{l}|\theta )}{C(x^{l})};\;C\left(x^{l}\right)=\int f\left(x^{l}|\theta \right)d\theta$$

if $\int f(x^{l};\theta )d\theta$ is finite. For comparison, the Bayes posteriori distribution is

$$f\left(\theta |x^{l}\right)=\frac{f\left(x^{l}|\theta \right)f\left(\theta \right)}{\int f\left(x^{l}|\theta \right)f\left(\theta \right)d\theta }.$$

If the support Θ of θ has finite area, (12) is just the Bayes predictor with uniform prior. If the support Θ of θ does not have finite area, we can get (12) as a limiting case when we take the limit of uniform distributions on finite $\Theta_{n}$ that converge towards Θ. This is the same way the ML estimator can be seen as a MAP estimator with uniform prior [46]. One can reasonably argue that if we have no further information about θ, a uniform distribution seems reasonable, and has indeed been used for MDL [47] as well as universal source coding ([10], Section 13.2). What the Normalized Likelihood Method does is simply extend this to the case when there is no proper uniform prior for θ.

The method was actually implicitly mentioned as a remark by Rissanen in ([48], Section 3.2), but to our knowledge was never further developed; the main contribution in this paper is to introduce the method as a practical method. From Rissanen we also know the coding distribution for $x_{n}$:

(13) $$f\left(x_{n+1}|x^{n}\right)=\int f\left(x_{n+1}|\theta \right)f_{x^{n}}\left(\theta \right)d\theta =\frac{C\left(x^{n+1}\right)}{C\left(x^{n}\right)}.$$

Let us assume $C(x^{n})$ becomes finite for n>1 (this is not always the case, often *n* needs to be larger). The total codelength can then be written as

(14) $$\begin{array}{cc} L(x^{l}) & =\sum_{i=1}^{l-1}-\log f\left(x_{i+1}|x^{i}\right)-\log f_{d}\left(x_{1}\right)\\ & =-\log C\left(x^{l}\right)+\log C\left(x^{2}\right)-\log f_{d}\left(x_{1}\right), \end{array}$$

where $f_{d}\left(x\right)$ is the default distribution, which for application in atypicality can be taken as the typical distribution. One might see this simply as a (generalized) Bayesian method. However, in general $C(x^{n})$ is not a valid probability, and as mentioned in ([9], Section 3.4) an improper prior cannot be used for Bayesian model selection. But when implemented sequentially, as indicated in (14) it does give a valid codelength, because of the principle of sequentiality, central to coding.

### 2.3. Examples

We will compare the different methods for a simple model. Assume our model is $N(0,\sigma^{2})$ with σ unknown. The likelihood function is $f\left(x^{n}|\sigma^{2}\right)=\frac{1}{{\left(2\pi \sigma^{2}\right)}^{n/2}}\exp\left(-\frac{1}{2\sigma^{2}}\sum_{i=1}^{n}x_{i}^{2}\right)$. For n=1 we have $\int_{0}^{\infty }f\left(x^{n}|\sigma^{2}\right)d\sigma^{2}=\infty$, but for n≥2

$$\begin{array}{cc} C\left(x^{n}\right) & =\int f\left(x^{n}|\sigma^{2}\right)d\sigma^{2}=\frac{1}{\pi^{\frac{n}{2}}2}\frac{\Gamma \left(\frac{n-2}{2}\right)}{{\left[n{\hat{\sigma^{2}}}_{n}\right]}^{\frac{n-2}{2}}} \end{array}$$

then

$$\begin{array}{cc} f_{\mathrm{nlm}}\left(x_{n+1}|x^{n}\right) & =\frac{\Gamma \left(\frac{n-1}{2}\right)}{\sqrt{\pi }\Gamma \left(\frac{n-2}{2}\right)}\frac{{\left[n{\hat{\sigma^{2}}}_{n}\right]}^{\frac{n-2}{2}}}{{\left[\left(n+1\right){\hat{\sigma^{2}}}_{n+1}\right]}^{\frac{n-1}{2}}} \end{array}$$

where ${\hat{\sigma^{2}}}_{n}=\frac{1}{n}\sum_{i=1}^{n}x_{i}^{2}$. Thus, for coding, the two first samples would be encoded with the default distribution, and after that the above distribution is used. For the SSM, we note that ${\hat{\sigma^{2}}}_{n}$ is a sufficient statistic for $\sigma^{2}$ and that $z=\frac{n}{\sigma^{2}}{\hat{\sigma^{2}}}_{n}∼\chi_{\left(n\right)}^{2}$, i.e., ${\hat{\sigma^{2}}}_{n}=s\left(z,\sigma^{2}\right)=\frac{\sigma^{2}}{n}z$, which we can be solved as $\sigma^{2}=r\left(z,{\hat{\sigma^{2}}}_{n}\right)=\frac{n}{z}{\hat{\sigma^{2}}}_{n}$, in the notation of (9)–(10). This is a transformation of the $\chi_{\left(n\right)}^{2}$ distribution which can be easily found as [6]

$$\begin{array}{cc} f_{x^{n}}\left(\sigma^{2}\right) & =\frac{{\left[n{\hat{\sigma^{2}}}_{n}\right]}^{\frac{n}{2}}}{2^{\frac{n}{2}}\Gamma \left(\frac{n}{2}\right){\left(\sigma^{2}\right)}^{\frac{n+2}{2}}}\exp\left\{-\frac{n}{2\sigma^{2}}{\hat{\sigma^{2}}}_{n}\right\}. \end{array}$$

Now we have

(15) $$\begin{array}{cc} f_{\mathrm{ssm}}\left(x_{n+1}|x^{n}\right) & =\int f\left(x_{n+1}|\sigma^{2}\right)f_{x^{n}}\left(\sigma^{2}\right)d\sigma^{2}\\ & =\frac{\Gamma \left(\frac{n+1}{2}\right)}{\sqrt{\pi }\Gamma \left(\frac{n}{2}\right)}\frac{{\left[n{\hat{\sigma^{2}}}_{n}\right]}^{\frac{n}{2}}}{{\left[\left(n+1\right){\hat{\sigma^{2}}}_{n+1}\right]}^{\frac{n+1}{2}}}. \end{array}$$

For comparison, the ordinary predictive MDL is

(16) $$\begin{array}{cc} f(x_{n+1}|x^{n}) & =\frac{1}{\sqrt{2\pi {\hat{\sigma^{2}}}_{n}}}\exp\left(-\frac{1}{2{\hat{\sigma^{2}}}_{n}}x_{n+1}^{2}\right) \end{array}$$

which is of a completely different form. To understand the difference, consider the codelength for $x_{2}$:

$$\begin{array}{ccc} L(x_{2}) & =\log\left(\frac{x_{1}^{2}+x_{2}^{2}}{|x_{1}|}\right)+\log\left(\frac{\sqrt{\pi }\Gamma \left(\frac{1}{2}\right)}{\Gamma (1)}\right) & \mathrm{SSM},\\ L(x_{2}) & =\frac{1}{2}\log\left(2\pi x_{1}^{2}\right)+\frac{x_{2}^{2}}{x_{1}^{2}} & \mathrm{predictive}\;\mathrm{MDL}. \end{array}$$

It can be seen that if $x_{1}$ is small and $x_{2}$ is large, the codelength for $x_{2}$ is going to be large. But in the sufficient statistic method this is strongly attenuated due to the log in front of the ratio. Figure 1 shows this quantitatively in the redundancy sense. The redundancy is the difference between the codelength using true and estimated distributions. As can be seen, the CDF of the ordinary predictive MDL redundancy has a long tail, and this is taken care of by SSM.

## 3. Scalar Signal Processing Methods

In the following we will derive MDL for various scalar signal processing methods. We can take inspiration from signal processing methods generally used for source coding, such as linear prediction and wavelets; however, the methods have to be modified for MDL, as we use lossless coding, not lossy coding. As often in signal processing, the models are a (deterministic) signal in Gaussian noise. In a previous paper we have also considered non-Gaussian models [7]. All proofs are in Appendices.

### 3.1. Iid Gaussian Case

A natural extension of the examples considered in Section 2.1 is $x_{n}∼N\left(\mu ,\sigma^{2}\right)$ with both μ and $\sigma^{2}$ unknown. Define ${\hat{\mu }}_{n}=\frac{1}{n}\sum_{i=1}^{n}x_{i}$ and $S_{n}^{2}=\frac{1}{n-1}\sum_{i=1}^{n}{\left(x_{i}-{\hat{\mu }}_{n}\right)}^{2}$. Then the sufficient statistic method is

(17) $$\begin{array}{cc} f(x_{n+1}|x^{n}) & =\sqrt{\frac{n}{\pi \left(n+1\right)}}\frac{\Gamma \left(\frac{n}{2}\right)}{\Gamma \left(\frac{n-1}{2}\right)}\\ & \times \frac{{\left[\left(n-1\right)S_{n}^{2}\right]}^{\frac{n-1}{2}}}{{\left[nS_{n+1}^{2}\right]}^{\frac{n}{2}}}. \end{array}$$

This is a special case of the vector Gaussian model considered later, so we will not provide a proof.

#### 3.1.1. Linear Transformations

The iid Gaussian case is a fundamental building block for other MDL methods. The idea is to find a linear transformation so that we can model the result as iid, and then use the iid Gaussian MDL. For example, in the vector case, suppose $x_{n}∼N\left(\mu ,\Sigma \right)$ is (temporally) iid, and let $y_{n}=Ax_{n}∼N\left(A\mu ,A\Sigma A^{T}\right)$. If we then assume that $A\Sigma A^{T}$ is diagonal, we can use the iid Gaussian MDL on each component. Similarly, in the scalar case, we can use a filter instead of a matrix. Because of (4) we need to require A to be orthonormal: For any input we then have $y_{n}^{T}y_{n}=x_{n}^{T}A^{T}Ax_{n}=x_{n}^{T}x_{n}$, and in particular $E\left[y_{n}^{T}y_{n}\right]=E\left[x_{n}^{T}x_{n}\right]$ independent of the actual Σ. We will see this approach in several cases in the following.

### 3.2. Linear Prediction

Linear prediction is a fundamental to random processes. Write

$$\begin{array}{cc} {\hat{x}}_{n+1|x^{n}} & =\overset{\infty }{\sum_{k=0}}w_{k}x_{n-k}\\ e_{n+1} & =x_{n+1}-{\hat{x}}_{n+1|x^{n}}. \end{array}$$

Then for most stationary random processes the resulting random process $\left\{e_{n}\right\}$ is uncorrelated, and hence in the Gaussian case, iid, by the Wold decomposition [6]. It is therefore a widely used method for source coding, e.g., [13]. In practical coding, a finite prediction order *M* is used,

$$\begin{array}{cc} {\hat{x}}_{n+1|x^{n}} & =\overset{M}{\sum_{k=1}}w_{k}x_{n-k+1},\;n\geq M \end{array}$$

Denote by τ the power of $\left\{e_{n}\right\}$. Consider the simplest case with M=1: There are two unknown parameters $\left(w_{1},\tau \right)$. However, the minimal sufficient statistic has dimension three [49]: $\left(\sum_{k=1}^{n}x_{k}^{2},\sum_{k=1}^{n-1}x_{k}^{2},\sum_{k=2}^{n}x_{k}x_{k-1}\right)$. Therefore, we cannot use SSM; and even if we could, the distribution of the sufficient statistic is not known in closed form [49]. We therefore turn to the NLM.

We assume that $e_{n+1}=x_{n+1}-{\hat{x}}_{n+1|x^{n}}$ is iid normally distributed with zero mean and variance τ,

(18) $$\begin{array}{cc} f(x^{n}|\tau ,w) & =\frac{1}{{\left(2\pi \tau \right)}^{(n-M)/2}}\\ & \times \exp\left(-\frac{1}{2\tau }\sum_{i=M+1}^{n}{\left[x_{i}-\sum_{k=1}^{M}w_{k}x_{i-k}\right]}^{2}\right). \end{array}$$

Define

$$\begin{array}{cc} {\hat{r}}_{\left(n\right)}\left(k\right) & =\sum_{i=M+1}^{n}x_{i}x_{i-k}. \end{array}$$

Then a simple calculation shows that

$$\begin{array}{cc} \sum_{i=M+1}^{n}e_{i}^{2} & ={\hat{r}}_{\left(n\right)}\left(0\right)-2w^{T}p_{\left(n\right)}+w^{T}R_{\left(n\right)}^{\left(M\right)}w \end{array}$$

where $w^{T}=\left[w_{1}\;w_{2}\;\cdots \;w_{M}\right]$, $p_{\left(n\right)}^{T}=\left[{\hat{r}}_{\left(n\right)}\left(1\right)\;{\hat{r}}_{\left(n\right)}\left(2\right)\;\cdots \;{\hat{r}}_{\left(n\right)}\left(M\right)\right]$,

(19) $$\begin{array}{cc} R_{\left(n\right)}^{\left(M\right)} & =\sum_{i=M+1}^{n}x_{i-M}^{i-1}{\left(x_{i-M}^{i-1}\right)}^{T} \end{array}$$

and $x_{i-M}^{i-1}=\left[x_{i-1},x_{i-2},\ldots ,x_{i-M}\right]$. Thus

$$\begin{array}{cc} f(x^{n}|\tau ,w) & =\frac{1}{{\left(2\pi \tau \right)}^{(n-M)/2}}\\ & \times \exp\left(-\frac{1}{2\tau }\left[{\hat{r}}_{\left(n\right)}\left(0\right)-2w^{T}p_{\left(n\right)}+w^{T}R_{\left(n\right)}^{\left(M\right)}w\right]\right) \end{array}$$

giving (see Appendix A)

$$\begin{array}{cc} C(x^{n}) & =\frac{1}{2{\left(\pi \right)}^{\frac{n-2M}{2}}\sqrt{\det\left(R_{\left(n\right)}\right)}}\frac{\Gamma \left(\frac{n-2M-2}{2}\right)}{{\left({\hat{\tau }}_{\left(n\right)}^{\left(M\right)}\right)}^{\frac{n-2M-2}{2}}} \end{array}$$

and

(20) $$\begin{array}{cc} f_{M}\left(x_{n+1}|x^{n}\right) & =\sqrt{\frac{\det\left(R_{\left(n\right)}^{\left(M\right)}\right)}{\det\left(R_{\left(n+1\right)}^{\left(M\right)}\right)}}\frac{\Gamma \left(\frac{n-2M-1}{2}\right)}{\Gamma \left(\frac{n-2M-2}{2}\right)}\\ & \times \frac{1}{\sqrt{\pi }}\frac{{\left({\hat{\tau }}_{\left(n\right)}^{\left(M\right)}\right)}^{\frac{n-2M-2}{2}}}{{\left({\hat{\tau }}_{\left(n+1\right)}^{\left(M\right)}\right)}^{\frac{n-2M-1}{2}}} \end{array}$$

with ${\hat{\tau }}_{\left(n\right)}^{\left(M\right)}={\hat{r}}_{\left(n\right)}\left(0\right)-p_{\left(n\right)}^{T}R_{\left(n\right)}^{-1}p_{\left(n\right)}$.

The Equation (20) is defined for n≥2M+2: The vector $x_{i-M}^{i-1}$ is defined for i≥M+1, and $R_{\left(n\right)}^{\left(M\right)}$ defined by (19) becomes full rank when the sum contains *M* terms. Before the order *M* linear predictor becomes defined, the data needs to be encoded with other methods. Since in atypicality we are not seeking to determine the model of data, just if a different model than the typical is better, we encode data with lower order linear predictors until the order *M* linear predictor becomes defined. So, the first sample is encoded with the default pdf. The second and third samples are encoded with the iid unknown variance coder (There is no issue in encoding some samples with SSM and others with NLM) (15). Then the order 1 linear predictor takes over, and so on.

### 3.3. Filterbanks and Wavelets

A popular approach to source coding is sub-band coding and wavelets [50,51,52]. The basic idea is to divide the signal into (perhaps overlapping) spectral sub-bands and then allocate different bitrates to each sub-band; the bitrate can be dependent on the power in the sub-band and auditory properties of the ear in for example audio coding. In MDL we need to do lossless coding, so this approach cannot be directly applied, but we can still use sub-band coding as explained in the following.

As we are doing lossless coding, we will only consider perfect reconstruction filterbanks [50,53]. Furthermore, in light of Section 3.1.1 we also consider only (normalized) orthogonal filterbanks [50,52].

The basic idea is that we split the signal into a variable number of sub-bands by putting the signal through the filterbank and downsampling. Then the output of each downsampled filter is coded with the iid Gaussian coder of Section 3.1 with an unknown mean and variance, which are specific to each sub-band. In order to understand how this works, consider a filterbank with two sub-bands. Assume that the signal is stationary zero mean Gaussian with power $\sigma^{2}$, and let the power at the output of sub-band 1 be $\sigma_{1}^{2}$ and of sub-band 2 be $\sigma_{2}^{2}$. Because the filterbank is orthogonal, we have $\sigma^{2}=\frac{1}{2}\left(\sigma_{1}^{2}+\sigma_{2}^{2}\right)$. To give some intuition to why a sub-band coder can give shorter codelengh, we use (8) to get the approximate codelengths

$$\begin{array}{cc} L_{\mathrm{direct}} & =\frac{l}{2}\log\left(\sigma^{2}\right)+\frac{l}{2}\left(\log2\pi +\log e\right)+\frac{1}{2}\log l\\ L_{\mathrm{filterbank}} & =\frac{l}{4}\log\left(\sigma_{1}^{2}\right)+\frac{l}{4}\left(\log2\pi +\log e\right)+\frac{1}{2}\log l\\ & +\frac{l}{4}\log\left(\sigma_{2}^{2}\right)+\frac{l}{4}\left(\log2\pi +\log e\right)+\frac{1}{2}\log l\\ & =\frac{l}{2}\log\left(\sqrt{\sigma_{1}^{2}\sigma_{2}^{2}}\right)+\frac{l}{2}\left(\log2\pi +\log e\right)+\log l. \end{array}$$

Since $\sqrt{\sigma_{1}^{2}\sigma_{2}^{2}}\leq \sigma^{2}$ (with equality only if $\sigma_{1}^{2}=\sigma_{2}^{2}$), the sub-band coder will result in shorter codelength for sufficiently large *l* if the signal is non-white.

The above analysis is a stationary analysis for long sequences. However, when considering shorter sequences, we also need to consider the transient. The main issue is that output power will deviate from the stationary value during the transient, and this will affect the estimated power $\hat{\sigma_{n}^{2}}$ used in the sequential MDL. The solution is to transmit to the receiver the input to the filterbank during the transient, and only use the output of the filterbank once the filters have been filled up. It is easy to see that the system is still perfect reconstruction: Using the received input to the filterbank, the receiver puts this through the analysis filterbank. It now has the total sequence produced by the analysis filterbank, and it can then put that through the reconstruction filterbank. When using multilevel filterbanks, this has to be done at each level.

We assume the decoder knows which filters are used and the maximum depth *D* used. In principle the encoder could now search over all trees of level at most *D*. The issue is that there are an astonishing large number of such trees; for example for D=4 there are 676 such trees. Instead of choosing the best, we can use the idea of the CTW [1,54,55] and weigh in each node: Suppose after passing a signal $x^{n}$ of an internal node *S* through low-pass and high-pass filters and downsampler, $x_{L}^{n/2}$ and $x_{H}^{n/2}$ are produced in the children nodes of *S*. The weighted probability of $x^{n}$ in the internal node *S* will be

$$\begin{array}{cc} f_{w}\left(x^{n}\right) & =\frac{1}{2}f\left(x^{n}\right)+\frac{1}{2}f_{w}\left(x_{L}^{n/2}\right)f_{w}\left(x_{H}^{n/2}\right) \end{array}$$

which is a good coding distribution for both a memoryless source and a source with memory [54,55].

## 4. Vector Case

We now assume that a vector sequence $x^{n}$, $x_{i}\in R^{M}$ is observed. The vector case allows for a more rich set of model and more interesting data discovery than the scalar case, for example atypical correlation between multiple sensors. It can also be applied to images [56], and to scalar data by dividing into blocks. That is in particular useful for the DFT, Section 4.4.

A specific concern is initialization. Applying sequential coding verbatim to the vector case means that the first vector $x_{1}$ needs to be encoded with the default coder, but this means the default coder influences the codelength too much. Instead we suggest to encode the first vector as a scalar signal using the scalar Gaussian coder (unknown variance→unknown mean/variance). That way only the first component of the first vector needs to be encoded with the default coder.

### 4.1. Vector Gaussian Case with Unknown Mean

First assume μ is unknown but Σ is given. We define $\mathrm{etr}\left(\cdots \right)=\exp\left(\mathrm{trace}\left(\cdots \right)\right)$ and we have

$$\begin{array}{cc} f\left(x^{n}|\mu \right) & =\frac{1}{\sqrt{{\left(2\pi \right)}^{kn}\det{\left(\Sigma \right)}^{n}}}\\ & \times \exp\left\{-\frac{1}{2}\sum_{i=1}^{n}{\left(x_{i}-\mu \right)}^{T}\Sigma^{-1}\left(x_{i}-\mu \right)\right\}. \end{array}$$

We first consider the NLM. By defining ${\hat{\mu }}_{n}=\frac{1}{n}\sum_{i=1}^{n}x_{i}$ and ${\hat{\Sigma }}_{n}=\sum_{i=1}^{n}x_{i}x_{i}$ (note that ${\hat{\Sigma }}_{n}$ is not the estimate of Σ) we have

$$\begin{array}{cc} C\left(x^{n}\right) & =\int f\left(x^{n}|\mu \right)d\mu \\ & =\frac{1}{\sqrt{{\left(2\pi \right)}^{kn}\det{\left(\Sigma \right)}^{n}}}\exp\left\{-\frac{1}{2}\sum_{i=1}^{n}x_{i}\Sigma^{-1}x_{i}\right\}\\ & \times \int \exp\left\{-\frac{n}{2}\mu^{T}\Sigma^{-1}\mu +n{\hat{\mu }}_{n}^{T}\Sigma^{-1}\mu \right\}d\mu \\ & =C\exp\left\{-\frac{1}{2}\sum_{i=1}^{n}\left(x_{i}\Sigma^{-1}x_{i}-{\hat{\mu }}_{n}^{T}\Sigma^{-1}{\hat{\mu }}_{n}\right)\right\}\\ & =C\mathrm{etr}\left\{-\frac{1}{2}\left({\hat{\Sigma }}_{n}-n{\hat{\mu }}_{n}{\hat{\mu }}_{n}^{T}\right)\Sigma^{-1}\right\} \end{array}$$

where $C=\frac{1}{\sqrt{{\left(2\pi \right)}^{k\left(n-1\right)}n^{k}\det{\left(\Sigma \right)}^{n-1}}}$, hence we can write

(21) $$\begin{array}{c} f\left(x_{n+1}|x^{n}\right)=\frac{C\left(x^{n+1}\right)}{C\left(x^{n}\right)}\\ =\sqrt{{\left(\frac{n}{n+1}\right)}^{k}}\frac{1}{\sqrt{{\left(2\pi \right)}^{k}\det\left(\Sigma \right)}}\\ \times \frac{\mathrm{etr}\left\{-\frac{1}{2}\left({\hat{\Sigma }}_{n+1}-\left(n+1\right){\hat{\mu }}_{n+1}{\hat{\mu }}_{n+1}^{T}\right)\Sigma^{-1}\right\}}{\mathrm{etr}\left\{-\frac{1}{2}\left({\hat{\Sigma }}_{n}-n{\hat{\mu }}_{n}{\hat{\mu }}_{n}^{T}\right)\Sigma^{-1}\right\}}. \end{array}$$

It turns out that in this case, the SSM gives the same result.

### 4.2. Vector Gaussian Case with Unknown Σ

Assume $x_{n}∼N\left(0,\Sigma \right)$ where the covariance matrix is unknown:

$$\begin{array}{cc} f\left(x^{n}|\Sigma \right) & =\frac{1}{\sqrt{{\left(2\pi \right)}^{kn}\det{\left(\Sigma \right)}^{n}}}\mathrm{etr}\left\{-\frac{1}{2}{\hat{\Sigma }}_{n}\Sigma^{-1}\right\} \end{array}$$

where ${\hat{\Sigma }}_{n}=\sum_{i=1}^{n}x_{i}x_{i}^{T}$.

In order to find the MDL using SSM, notice that we can write

$$x_{n}=Sz_{n},\;z_{n}∼N\left(0,I\right)$$

where $S=\Sigma^{\frac{1}{2}}$, that is S is some matrix that satisfies $SS^{T}=\Sigma$. A sufficient statistic for Σ is

$${\hat{\Sigma }}_{n}=\sum_{i=1}^{n}x_{i}x_{i}^{T}=S\sum_{i=1}^{n}z_{i}z_{i}^{T}S^{T}\overset{\mathrm{def}}{=}SUS^{T}.$$

Let ${\hat{S}}_{n}={\hat{\Sigma }}_{n}^{\frac{1}{2}}=SU^{\frac{1}{2}}$. Then we can solve $S={\hat{S}}_{n}U^{-\frac{1}{2}}$ and $\Sigma ={\hat{S}}_{n}U^{-1}{\hat{S}}_{n}^{T}$. Since $U^{-1}$ has Inverse-Wishart distribution $U^{-1}∼W_{M}^{-1}\left(I,n\right)$, one can write $\Sigma ∼W_{M}^{-1}\left({\hat{\Sigma }}_{n},n\right)$. Using this distribution we calculate in Appendix B that

(22) $$\begin{array}{cc} f\left(x_{n+1}|x^{n}\right) & =\frac{1}{\pi^{\frac{M}{2}}}\frac{\det{\left({\hat{\Sigma }}_{n}\right)}^{\frac{n}{2}}}{\det{\left({\hat{\Sigma }}_{n+1}\right)}^{\frac{n+1}{2}}}\frac{\Gamma_{M}\left(\frac{n+1}{2}\right)}{\Gamma_{M}\left(\frac{n}{2}\right)} \end{array}$$

where $\Gamma_{M}$ is the multivariate gamma function [57].

On the other hand, using the normalized likelihood method we have

$$\begin{array}{cc} C\left(x^{n}\right) & =\frac{\Gamma_{M}\left(\frac{n}{2}-\frac{M+1}{2}\right)}{2^{\frac{M\left(M+1\right)}{2}}\pi^{\frac{kn}{2}}\det{\left({\hat{\Sigma }}_{n}\right)}^{\frac{n}{2}-\frac{M+1}{2}}}, \end{array}$$

from which

(23) $$\begin{array}{cc} f\left(x_{n+1}|x^{n}\right) & =\frac{C\left(x^{n+1}\right)}{C\left(x^{n}\right)}\\ & =\frac{1}{\pi^{\frac{k}{2}}}\frac{\det{\left({\hat{\Sigma }}_{n}\right)}^{\frac{n}{2}-\frac{M+1}{2}}}{\det{\left({\hat{\Sigma }}_{n+1}\right)}^{\frac{n}{2}-\frac{M}{2}}}\frac{\Gamma_{M}\left(\frac{n}{2}-\frac{M}{2}\right)}{\Gamma_{M}\left(\frac{n}{2}-\frac{M+1}{2}\right)}. \end{array}$$

### 4.3. Vector Gaussian Case with Unknown Mean and Σ

Assume $x_{n}∼N\left(\mu ,\Sigma \right)$ where both mean and covariance matrix are unknown:

$$\begin{array}{cc} f\left(x^{n}|\mu ,\Sigma \right) & =\frac{1}{\sqrt{{\left(2\pi \right)}^{Mn}\det{\left(\Sigma \right)}^{n}}}\\ & \times \exp\left\{-\frac{1}{2}\sum_{i=1}^{n}{\left(x_{i}-\mu \right)}^{T}\Sigma^{-1}\left(x_{i}-\mu \right)\right\}. \end{array}$$

It is well-known [46] that sufficient statistics are ${\hat{\mu }}_{n}=\frac{1}{n}\sum_{i=1}^{n}x_{i}$ and ${\hat{\Sigma }}_{n}=\left(n-1\right)S_{n}=\sum_{i=1}^{n}\left(x_{i}-{\hat{\mu }}_{n}\right){\left(x_{i}-{\hat{\mu }}_{n}\right)}^{T}$. Let S be a square root of Σ, i.e., $SS^{T}=\Sigma$. We can then write

$$\begin{array}{cc} {\hat{\mu }}_{n} & =\mu +\frac{1}{\sqrt{n}}Sz\\ {\hat{\Sigma }}_{n} & =SUS^{T} \end{array}$$

where $z∼N\left(0,I\right)$ and $U∼W_{M}\left(I,n-1\right)$, z and U are independent, and $W_{M}$ is the Wishart distribution. We solve the second equation with respect to S as in Section 4.2 and the first with respect to μ, to get

$$\begin{array}{cc} \Sigma & ={\hat{S}}_{n}U^{-1}{\hat{S}}_{n}^{T}∼W_{M}^{-1}\left({\hat{\Sigma }}_{n},n-1\right)\\ \mu & ={\hat{\mu }}_{n}-\frac{1}{\sqrt{n}}Sz={\hat{\mu }}_{n}-\frac{1}{\sqrt{n}}{\hat{S}}_{n}U^{-\frac{1}{2}}z∼N\left({\hat{\mu }}_{n},\frac{1}{n}\Sigma \right) \end{array}$$

where ${\hat{S}}_{n}$ is a square root of ${\hat{\Sigma }}_{n}$. We can explicitly write the distributions as

$$\begin{array}{cc} f_{x^{n}}\left(\mu |\Sigma \right) & =\sqrt{\frac{n^{M}}{{\left(2\pi \right)}^{M}\det\left(\Sigma \right)}}\exp\left\{-\frac{n}{2}{\left(\mu -{\hat{\mu }}_{n}\right)}^{T}\Sigma^{-1}\left(\mu -{\hat{\mu }}_{n}\right)\right\}\\ f_{x^{n}}\left(\Sigma \right) & =\frac{\det{\left({\hat{\Sigma }}_{n}\right)}^{\frac{n-1}{2}}}{2^{\frac{M\left(n-1\right)}{2}}\Gamma_{M}\left(\frac{n-1}{2}\right)}\det{\left(\Sigma \right)}^{-\frac{n+M}{2}}\mathrm{etr}\left\{-\frac{1}{2}{\hat{\Sigma }}_{n}\Sigma^{-1}\right\}. \end{array}$$

Using these distributions, in Appendix C we calculate

$$\begin{array}{cc} f\left(x_{n+1}|x^{n}\right) & =\frac{1}{\pi^{\frac{M}{2}}}\sqrt{{\left(\frac{n}{n+1}\right)}^{M}}\frac{\det{\left({\hat{\Sigma }}_{n}\right)}^{\frac{n-1}{2}}}{\det{\left({\hat{\Sigma }}_{n+1}\right)}^{\frac{n}{2}}}\frac{\Gamma_{M}\left(\frac{n}{2}\right)}{\Gamma_{M}\left(\frac{n-1}{2}\right)} \end{array}$$

and for NLM

$$\begin{array}{cc} f\left(x_{n+1}|x^{n}\right) & =\frac{1}{\pi^{\frac{M}{2}}}\sqrt{{\left(\frac{n}{n+1}\right)}^{M}}\frac{\det{\left({\hat{\Sigma }}_{n}\right)}^{\frac{n-1}{2}-\frac{M+1}{2}}}{\det{\left({\hat{\Sigma }}_{n+1}\right)}^{\frac{n}{2}-\frac{M+1}{2}}}\\ & \times \frac{\Gamma_{M}\left(\frac{n-M-1}{2}\right)}{\Gamma_{M}\left(\frac{n-M-2}{2}\right)}. \end{array}$$

These are very similar to the case of known mean, Section 4.2. We require one more sample before the distributions become well-defined, and $\Sigma_{n}$ is defined differently.

### 4.4. Sparsity and DFT

We can specify a general method as follows. Let Φ be an orthonormal basis of $R^{M}$ and write the signal model as

$$x_{n}=\sum_{i=1}^{N}\left(A_{i}+s_{i,n}\right)\phi_{j(i)}+w_{n}.$$

Here *N* is the number of basis vectors used, and j(i),i=1,…,N their indices. The signal $s_{i,n}$ is iid $N(0,\sigma_{i})$, the noise $w_{n}$ iid $N(0,\sigma^{2}I)$, and $A_{i},\sigma_{i}^{2},\sigma^{2}$ are unknown. If we let $y_{n}=\Phi^{T}x_{n}$ and *J* the indices of the signal components then

$$\begin{array}{cc} y_{j(i),n} & =A_{i}+s_{i,n}+w_{j(i),n}=A_{i}+{\tilde{s}}_{i,n},\;j\left(i\right)\in J\\ y_{j,n} & =w_{j,n},\;j\notin J. \end{array}$$

Thus the $y_{j(i),n}$ can be encoded with the scalar Gaussian encoder of Section 3.1, while the $y_{j,n}$ can be encoded with a vector Gaussian encoder for $N(0,\sigma^{2}I_{M-N})$ using the following equation that is achieved using the SSM:

$$\begin{array}{cc} f\left(w_{n+1}|w^{n}\right) & =\frac{1}{\pi^{\frac{\left(M-N\right)}{2}}}\frac{\Gamma \left(\frac{\left(M-N\right)\left(n+1\right)}{2}\right)}{\Gamma \left(\frac{\left(M-N\right)n}{2}\right)}\\ & \times \frac{{\left[n{\hat{\tau }}_{n}\right]}^{\frac{\left(M-N\right)n}{2}}}{{\left[\left(n+1\right){\hat{\tau }}_{n+1}\right]}^{\frac{\left(M-N\right)\left(n+1\right)}{2}}} \end{array}$$

where ${\hat{\tau }}_{n}=\frac{1}{n}\sum_{i=1}^{n}w_{i}^{T}w_{i}$. Now we need to choose which coefficients j(i) to choose as signal components and inform the decoder. The set *J* can be communicated to the decoder by sending a sequence of 0,1 encoded with the universal encoder of ([10], Section 13.2) with $MH\left(\frac{N}{M}\right)+\frac{1}{2}\log M$ bits. The optimum set can in general only be found by trying all sets *J* and choosing the one with shortest codelength, which is infeasible. A heuristic approach is to find the *N* components with maximum power when calculated over the whole blocklength *l* (the decoder does not need to know how *J* was chosen, only what *J* is, it is therefore fine to use the power at the end of the block). What still remains is how to choose *N*. It seems computationally feasible to start with N=1 and then increase *N* by 1 until the codelength no longer decreases, since most of the calculations for *N* can be reused for N+1.

We can apply this in particular when Φ is a DFT matrix. In light of Section 3.1.1 we need to use the normalized form of the DFT. The complication is that the output is complex, i.e., the *M* real inputs result in *M* complex outputs, or 2M real outputs. Therefore, care has to be taken with the symmetry properties of the output. Another option is to use DCT instead, which is well-developed and commonly used for compression.

## 5. Experimental Results

### 5.1. Transient Detection Using Hydrophone Recordings

As an example of the application of atypicality, we will consider transient detection [28]. In transient detection, a sensor records a signal that is pure noise most of the time, and the task is to find the sections of the signal that are not noise. In our terminology, the typical signal is noise, and the task is to find the atypical parts.

As data we used hydrophone recordings from a sensor in the Hawaiian waters outside Oahu, the Station ALOHA Cabled Observatory (ACO) [58]. The data used for this paper were collected (with sampling freuquency of 96 kHz which was then downsampled to 8 kHz) during a proof module phase of the project conducted between February 2007 and October 2008. The data was pre-processed by differentiation (y[n]=x[n]−x[n−1]) to remove a non-informative mean component.

The principal goal of this two years of data is to locate whale vocalization. Fin (22 m, up to 80 tons) and sei (12–18 m, up to 24.6 tons) whales are known by means of visual and acoustic surveys to be present in the Hawaiian Islands during winter and spring months, but migration patterns in Hawaii are poorly understood [58].

Ground truth has been established by manual detection, which is achieved using visual inspection of spectrogram by a human operator. 24 h of manual detections for both the 20 Hz and the 20–35 Hz variable calls were recorded for each the following dates (randomly chosen): 1 March 2007, 17 November 2007, 29 May 2008, 22 August 2008, 4 September 2008 and 9 February 2008 [58].

In order to analyze the performance of different detectors on such a data, first the measures ’Precision’ and ’Recall’ are defined as below

$$\begin{array}{cc} \mathrm{Recall} & =\frac{\mathrm{number}\;\mathrm{of}\;\mathrm{correct}\;\mathrm{detection}s}{\mathrm{total}\;\mathrm{number}\;\mathrm{of}\;\mathrm{manual}\;\mathrm{detections}}\\ \mathrm{Precision} & =\frac{\mathrm{number}\;\mathrm{of}\;\mathrm{correct}\;\mathrm{detections}}{\mathrm{total}\;\mathrm{number}\;\mathrm{of}\;\mathrm{algorithm}\;\mathrm{detections}} \end{array}$$

where Recall measures the probability of correctly obtained vocalizations over expected number of detections and Precision measures the probability of correctly detected vocalizations obtained by the detector. The Precision versus Recall curve show the detectors ability to obtain vocalizations as well as the accuracy of these detections [58].

In order to compare our atypicality method with alternative approaches in transient detection, we compare its performance with Variable Threshold Page (VTP) which outperforms other similar methods in detection of non-trivial signals [31].

For the atypicality approach, we need a typical and an atypical coder. The typical signal is pure noise, which, however, is not necessarily white: It consists of background noise, wave motion, wind and rain. We therefore used a linear predictive coder. The order of the linear predictive coder was globally set to 10 as a compromise between performance and computational speed. An order above 10 showed no significant decrease in codelength, while increasing computation time. The prediction coefficients were estimated for each 5 min segment of data. It seems unreasonable to expect the prediction coefficients to be globally constant due to for example variations in weather, but over short length segments they can be expected to be constant. Of course, a 5 min segment could contain atypical data and that would result in incorrect typical prediction coefficients. However, for this particular data we know (or assume) that atypical segments are of very short duration, and therefore will affect the estimated coefficients very little. This cannot be used for general data sets, only for data sets where there is a prior knowledge (or assumption) that atypical data are rare and short. Otherwise the typical coder should be trained on data known to be typical as in [1] or by using unsupervised atypicality, which we are developing for a future paper.

For the atypical coder, we implemented all the scalar methods of Section 3 in addition to the DFT, Section 4.4, with optimization over blocklength. Let $X\left(n,l\right)=\left(x_{n},\ldots ,x_{n+l-1}\right)$ be a subsequence of length *l* to be tested for atypicality, and suppose $L_{T}\left(X(n,l)\right)$ and $L_{A}\left(X(n,l)\right)$ are the typical codelength and atypical codelength of sequence X(n,l), respectively. Note that $L_{A}\left(X(n,l)\right)=-\log\left(f(X(n,l))\right)+\log^{*}l$ where *f* is any encoder of Section 3 and Section 4.4, and $\log^{*}l$ is the number of bits to tell the decoder the length of the atypical subsequence, as discussed in Section 1.1, see also [1,59]. Then for every sample of data we calculate

(24) $$\begin{array}{cc} \Delta L(n) & =\max_{l}\left\{L_{T}\left(X\left(n,l\right)\right)-L_{A}\left(X\left(n,l\right)\right)\right\} \end{array}$$

and the atypicality criterion would be ΔL(n)>τ for some threshold (which does not need to be chosen prior to running the algorithm, since the larger ΔL(n) is the more atypical). Please note that the threshold τ can be seen as the length of the header the encoder uses to tell the decoder an atypical sequence is next. Calculating ΔL(n) requires examining every subsequence (perhaps up to a maximum length). Because the coders (e.g., (5)) are recursive, we can efficiently calculate $L_{A}\left(X(n,l+1)\right)$ from $L_{A}\left(X(n,l)\right)$, so the complexity is not prohibitive. Still, for a large dataset (i.e., big data), direct implementation of atypicality search is too computationally complex; so instead, similar to [59] we propose a tree-structured searching algorithm in which discovery of atypical sequence (in this case, whale vocalizations) can be performed in different stages. First in coarse search, a tree-structured division of data is considered such that at each level *i*, data is divided into non-overlapping blocks of length $2^{i}$, then for each block typical and atypical codelengths are compared. Obviously due to non-overlapping division some atypical sequences are missed, and the worse case is if an atypical sequence of length *l* is divided equally into two consecutive non-overlapping blocks of length $2^{i}$. However, each of these sequences of length l/2 might be detected at the level i−1. The issue is that the complexity penalty per sample from (8) is about $\frac{\frac{k}{2}\log l}{l}$, which is decreasing in *l*. Thus, a sequence of length *l* may be atypical, but each of the length $\frac{l}{2}$ halves may not be. This can be compensated by repeating every block once and encoding this double length block. By experimentation we have found that this gives a very low chance of missing an atypical subsequence. On the other hand, it does give false positives, because an exactly repeated block clearly has a strong (false) pattern. This is not a big issue, as these false positives are eliminated during the next stage.

**Figure 2:**
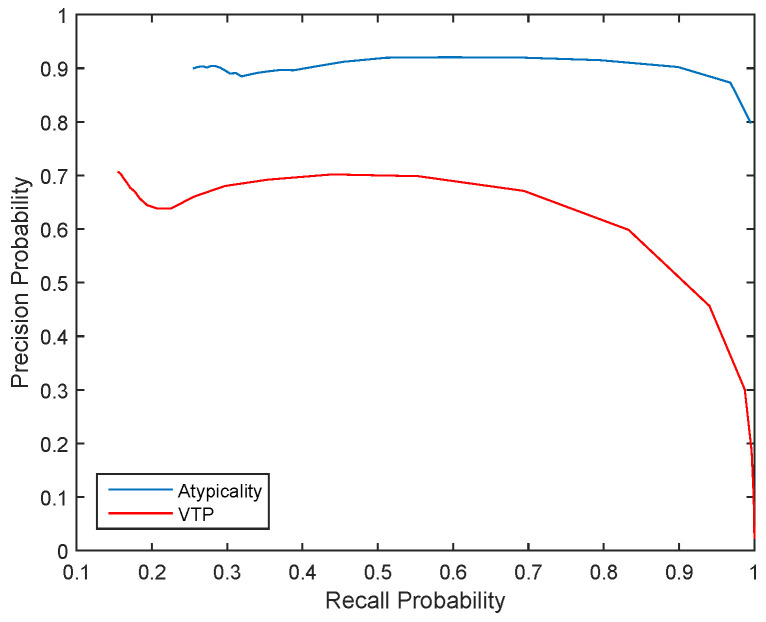
Precision vs. Recall probability for all six days that manual detections are available.

After the coarse search, the next stage is fine search, in which the blocks flagged by coarse search are expanded and every subsequence of this expanded block is tested in an exhaustive search, which eliminates false positives. The final stage is segmentation, where the exact start and end point of atypical sequences are determined by minimizing the total codelength of the whole sequence of data. Figure 2 shows Precision vs. Recall curve for both atypicality and VTP.

### 5.2. Anomaly Detection Using Holter Monitoring Data

As another example of atypicality application, we consider an anomaly detection problem. We consider data obtained by Holter Monitoring, i.e., a continuous tape recording of a patient’s ECG for 24 h. We use the MIT-BIH Normal Sinus Rhythm Database (nsrdb) which is provided by PhysioNet [60]. Even though the subjects included in this database were found to have had no significant persistent arrhythmias, there still existed arrhythmic beats and patterns to look for [60]. We apply atypicality to find interesting parts of the the dataset.

**Figure 3:**
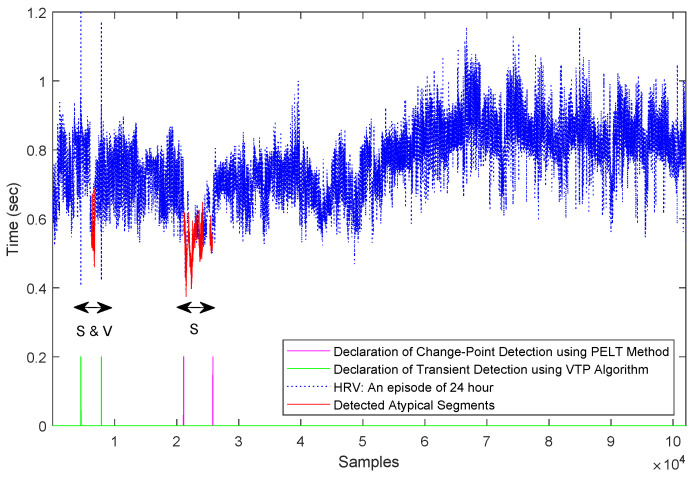
Detected atypical segments of Holter Monitoring heart rate variability (HRV): “S” stands for supraventricular arrhythmia and “V” stands for ventricular contraction based on annotation provided by PhysioNet [60].

Since the data is assumed to be ‘Normal Sinus Rhythm’, a Gaussian model with unknown mean and variance is assumed for the typical data. For atypical encoding, we used the same methodology as in the previous section. As can be seen in the Figure 3, atypicality as an anomaly detector was able to find two major atypical segments, both of which contained multiple supraventricular beats and ventricular contraction (provided by HRV annotation files, PhysioNet [60]). Based on the data annotation these two segments were the only fractions in the data that contained abnormal beats and rhythms, which shows the efficacy of the atypicality framework. For comparison we included VTP as a transient detection method and the pruned exact linear time (PELT) method [37] as a change-point detection algorithm. As can be seen, VTP and PELT detected only one of the anomalous segments, while atypicality detected both.

## 6. Conclusions

Atypicality is a method for finding rare, interesting snippets in big data. It can be used for anomaly detection, data mining, transient detection, and knowledge extraction among other things. The current paper extended atypicality to real-valued data. It is important here to notice that discrete-valued and real-valued atypicality is one theory. Atypicality can therefore be used on data that are of mixed type. One advantage of atypicality is that it directly applies to sequences of variable length. Another advantage is that there is only one parameter that regulates atypicality, the single threshold parameter τ, which has the concrete meaning of the logarithm of the frequency of atypical sequences. This contrasts with other methods that have multiple parameters.

Atypicality becomes really interesting in combination with machine learning. First, atypicality can be used to find what is not learned in machine learning. Second, for many data sets, machine learning is needed to find the typical coder. In the experiments in this paper, we did not need machine learning because the typical data was pure noise. But in many other types of data, e.g., ECG (electrocardiogram), ‘normal’ data is highly complex, and the optimum coder has to be learned with machine learning. This is a topic for future research. 

## Appendix A. Linear Prediction

We showed

$$\begin{array}{cc} f(x^{n}|\tau ,w) & =\frac{1}{{\left(2\pi \tau \right)}^{(n-M)/2}}\\ & \times \exp\left(-\frac{1}{2\tau }\left[{\hat{r}}_{\left(n\right)}\left(0\right)-2w^{T}p_{\left(n\right)}+w^{T}R_{\left(n\right)}^{\left(M\right)}w\right]\right), \end{array}$$

therefore using NLM we have

$$\begin{array}{cc} C(x^{n}) & =\int \int f(x^{n}|\tau ,w)dwd\tau \\ & =A\int_{\tau }\tau^{-\frac{\left(n-M\right)}{2}}\exp\left\{-\frac{{\hat{r}}_{\left(n\right)}\left(0\right)}{2\tau }\right\}e_{1}\left(\tau \right)d\tau \end{array}$$

where $A=\frac{1}{{\left(2\pi \right)}^{\left(n-M\right)/2}}$ and $e_{1}\left(\tau \right)=\int_{w}\exp\left\{-\frac{1}{2\tau }\left[w^{T}R_{\left(n\right)}w-2p_{\left(n\right)}^{T}w\right]\right\}dw$. Hence

$$\begin{array}{cc} C(x^{n}) & =B\int_{\tau }\tau^{-\frac{n-2M}{2}}\exp\left\{-\frac{1}{2\tau }\left[{\hat{r}}_{\left(n\right)}\left(0\right)-p_{\left(n\right)}^{T}R_{\left(n\right)}^{-1}p_{\left(n\right)}\right]\right\}d\tau \\ & =B\int_{\tau }\tau^{-\frac{n-2M}{2}}\exp\left\{-\frac{1}{2\tau }{\hat{\tau }}_{\left(n\right)}^{\left(M\right)}\right\}d\tau \\ & =\frac{1}{2{\left(\pi \right)}^{\frac{n-2M}{2}}\sqrt{\det\left(R_{\left(n\right)}\right)}}\frac{\Gamma \left(\frac{n-2M-2}{2}\right)}{{\left({\hat{\tau }}_{\left(n\right)}^{\left(M\right)}\right)}^{\frac{n-2M-2}{2}}} \end{array}$$

where $B=\frac{1}{{\left(2\pi \right)}^{\left(n-2M\right)/2}\sqrt{\det\left(R_{\left(n\right)}\right)}}$.

## Appendix B. Vector Gaussian Case: Unknown Σ

We showed that Σ has Inverse-Wishart distribution $\Sigma ∼W_{M}^{-1}\left({\hat{\Sigma }}_{n},n\right)$ where ${\hat{\Sigma }}_{n}=\sum_{i=1}^{n}x_{i}x_{i}$, hence

$$\begin{array}{cc} f_{x^{n}}\left(\Sigma \right) & =\frac{\det{\left({\hat{\Sigma }}_{n}\right)}^{\frac{n}{2}}}{2^{\frac{nM}{2}}\Gamma_{M}\left(\frac{n}{2}\right)}\det{\left(\Sigma \right)}^{-\frac{n+M+1}{2}}\mathrm{etr}\left\{-\frac{1}{2}{\hat{\Sigma }}_{n}\Sigma^{-1}\right\}, \end{array}$$

and since

$$\begin{array}{cc} f\left(x_{n+1}|\Sigma \right) & =\frac{1}{\sqrt{{\left(2\pi \right)}^{M}\det\left(\Sigma \right)}}\mathrm{etr}\left\{-\frac{1}{2}\left({\hat{\Sigma }}_{n+1}-{\hat{\Sigma }}_{n}\right)\Sigma^{-1}\right\}, \end{array}$$

therefore we have

$$\begin{array}{cc} f\left(x_{n+1}|x^{n}\right) & =\int_{\Sigma >0}f\left(x_{n+1}|\Sigma \right)f_{x^{n}}\left(\Sigma \right)d\Sigma \\ & =C\int_{\Sigma >0}\det{\left(\Sigma \right)}^{-\frac{n+M+2}{2}}\mathrm{etr}\left\{-\frac{1}{2}{\hat{\Sigma }}_{n+1}\Sigma^{-1}\right\}d\Sigma \\ & \overset{\left(A\right)}{=}C\int_{Y>0}\det{\left(Y\right)}^{\frac{n}{2}-\frac{M}{2}}\mathrm{etr}\left\{-\frac{1}{2}{\hat{\Sigma }}_{n+1}Y\right\}dY\\ & =D\int_{V>0}\det{\left(V\right)}^{\frac{n}{2}-\frac{M}{2}}\mathrm{etr}\left\{-V\right\}dV\\ & \overset{\left(B\right)}{=}D\int_{V>0}\det{\left(V\right)}^{\frac{n+1}{2}-\frac{M+1}{2}}\mathrm{etr}\left\{-V\right\}dV\\ & =\frac{1}{\pi^{\frac{M}{2}}}\frac{\det{\left({\hat{\Sigma }}_{n}\right)}^{\frac{n}{2}}}{\det{\left({\hat{\Sigma }}_{n+1}\right)}^{\frac{n+1}{2}}}\frac{\Gamma_{M}\left(\frac{n+1}{2}\right)}{\Gamma_{M}\left(\frac{n}{2}\right)} \end{array}$$

where $C=\frac{\det{\left({\hat{\Sigma }}_{n}\right)}^{\frac{n}{2}}}{2^{\frac{M\left(n+1\right)}{2}}\Gamma_{M}\left(\frac{n}{2}\right)\pi^{\frac{M}{2}}}$ and $D=\frac{\det{\left({\hat{\Sigma }}_{n}\right)}^{\frac{n}{2}}}{\det{\left({\hat{\Sigma }}_{n+1}\right)}^{\frac{n+1}{2}}}\frac{1}{\Gamma_{M}\left(\frac{n}{2}\right)\pi^{\frac{M}{2}}}$, and in Equations (A) and (B) we changed the variable $\Sigma =Y^{-1}$ and $Y=2{\hat{\Sigma }}_{n}^{-\frac{1}{2}}V{\hat{\Sigma }}_{n}^{-\frac{1}{2}}$ respectively and $\Gamma_{m}\left(a\right)=\int_{V>0}\det{\left(V\right)}^{a-\frac{\left(m+1\right)}{2}}\mathrm{etr}\left\{-V\right\}dV$ is the multivariate Gamma function.

## Appendix C. Vector Gaussian Case: Unknown Mean and Σ

We showed that $\mu ∼N\left({\hat{\mu }}_{n},\frac{1}{n}\Sigma \right)$ and $\Sigma ∼W_{M}^{-1}\left({\hat{\Sigma }}_{n},n-1\right)$ where ${\hat{\mu }}_{n}=\frac{1}{n}\sum_{i=1}^{n}x_{i}$ and ${\hat{\Sigma }}_{n}=\sum_{i=1}^{n}\left(x_{i}-{\hat{\mu }}_{n}\right){\left(x_{i}-{\hat{\mu }}_{n}\right)}^{T}$. Now using Bayes we can write the joint pdf as $f_{x^{n}}\left(\mu ,\Sigma \right)=f_{x^{n}}\left(\mu |\Sigma \right)f_{x^{n}}\left(\Sigma \right)$. Define $A\overset{\mathrm{def}}{=}f\left(x_{n+1}|x^{n}\right)=\int_{\Sigma >0}\int f\left(x_{n+1}|\mu ,\Sigma \right)f_{x^{n}}\left(\mu ,\Sigma \right)d\mu d\Sigma$,

$$\begin{array}{cc} A & =B\int_{\Sigma >0}\det{\left(\Sigma \right)}^{-\frac{n+M+2}{2}}e_{1}\left(\Sigma \right)e_{2}\left(\Sigma \right)d\Sigma \end{array}$$

where

$$\begin{array}{cc} e_{1}\left(\Sigma \right) & =\mathrm{etr}\left\{-\frac{1}{2}\left({\hat{\Sigma }}_{n}+n{\hat{\mu }}_{n}{\hat{\mu }}_{n}^{T}+x_{n+1}x_{n+1}^{T}\right)\Sigma^{-1}\right\}\\ e_{2}\left(\Sigma \right) & =\int \exp\left\{-\frac{n+1}{2}\left[\mu^{T}\Sigma^{-1}\mu -2{\hat{\mu }}_{n+1}\Sigma^{-1}\mu \right]\right\}d\mu \\ & =\sqrt{\frac{{\left(2\pi \right)}^{M}\det\left(\Sigma \right)}{{\left(n+1\right)}^{M}}}\exp\left\{\frac{n+1}{2}{\hat{\mu }}_{n+1}^{T}\Sigma^{-1}{\hat{\mu }}_{n+1}\right\}\\ B & =\frac{\det{\left({\hat{\Sigma }}_{n}\right)}^{\frac{n-1}{2}}}{\Gamma_{M}\left(\frac{n-1}{2}\right)}\frac{n^{\frac{M}{2}}}{2^{\frac{M\left(n-1\right)}{2}}{\left(2\pi \right)}^{M}}. \end{array}$$

Now since ${\hat{\Sigma }}_{n+1}={\hat{\Sigma }}_{n}+n{\hat{\mu }}_{n}{\hat{\mu }}_{n}^{T}+x_{n+1}x_{n+1}^{T}-\left(n+1\right){\hat{\mu }}_{n+1}{\hat{\mu }}_{n+1}^{T}$, by defining $C\overset{\mathrm{def}}{=}B\sqrt{\frac{{\left(2\pi \right)}^{M}}{{\left(n+1\right)}^{M}}}=\sqrt{{\left(\frac{n}{n+1}\right)}^{M}}\frac{\det{\left({\hat{\Sigma }}_{n}\right)}^{\frac{n-1}{2}}}{\Gamma_{M}\left(\frac{n-1}{2}\right)}\frac{1}{2^{\frac{M\left(n-1\right)}{2}}{\left(2\pi \right)}^{\frac{M}{2}}}$ we can write

$$\begin{array}{cc} A & =C\int_{\Sigma >0}\det{\left(\Sigma \right)}^{-\frac{n+M+1}{2}}\mathrm{etr}\left\{-\frac{1}{2}{\hat{\Sigma }}_{n+1}\Sigma^{-1}\right\}d\Sigma \\ & =C\int_{Y>0}\det{\left(Y\right)}^{\frac{n}{2}-\frac{M+1}{2}}\mathrm{etr}\left\{-\frac{1}{2}{\hat{\Sigma }}_{n+1}Y\right\}dY\\ & =C\frac{2^{\frac{Mn}{2}}}{\det{\left({\hat{\Sigma }}_{n+1}\right)}^{\frac{n}{2}}}\int_{V>0}\det{\left(V\right)}^{\frac{n}{2}-\frac{M+1}{2}}\mathrm{etr}\left\{-V\right\}dV\\ & =\frac{1}{\pi^{\frac{M}{2}}}\sqrt{{\left(\frac{n}{n+1}\right)}^{M}}\frac{\det{\left({\hat{\Sigma }}_{n}\right)}^{\frac{n-1}{2}}}{\det{\left({\hat{\Sigma }}_{n+1}\right)}^{\frac{n}{2}}}\frac{\Gamma_{M}\left(\frac{n}{2}\right)}{\Gamma_{M}\left(\frac{n-1}{2}\right)}. \end{array}$$
